# Effects of gelatin type and concentration on the preparation and properties of freeze-dried fish oil powders

**DOI:** 10.1038/s41538-024-00251-4

**Published:** 2024-02-03

**Authors:** Mengyang Yang, Jiawei Peng, Cuiping Shi, Ye Zi, Yulu Zheng, Xichang Wang, Jian Zhong

**Affiliations:** 1 Medical Food Laboratory, Shanghai Key Laboratory of Pediatric Gastroenterology and Nutrition, Shanghai Institute for Pediatric Research, Xinhua Hospital, Shanghai Jiao Tong University School of Medicine, Shanghai, 200092 China; 2 National R&D Branch Center for Freshwater Aquatic Products Processing Technology (Shanghai), Integrated Scientific Research Base on Comprehensive Utilization Technology for By-Products of Aquatic Product Processing, Ministry of Agriculture and Rural Affairs of the People’s Republic of China, Shanghai Engineering Research Center of Aquatic-Product Processing and Preservation, College of Food Science & Technology, Shanghai Ocean University, Shanghai, 201306 China; 3 Department of Clinical Nutrition, College of Health Science and Technology, Shanghai Jiao Tong University School of Medicine, Shanghai, 200135 China; 4 Marine Biomedical Science and Technology Innovation Platform of Lingang Special Area, Shanghai, 201306 China

**Keywords:** Structural properties, Chemical engineering

## Abstract

The effects of gelatin type (porcine skin gelatin, PSG; bovine skin gelatin, BSG; fish gelatin, FG; or cold-water fish skin gelatin, CFG) and concentration on the preparation and properties of fish oil powders were investigated in this work. The oil powders were prepared using the combination method of gelatin-sodium hexametaphosphate complex coacervation with starch sodium octenyl succinate (SSOS)-aided freeze-drying. Compared with the other gelatins, CFG—with an unobvious isoelectric point, a lower molecular weight, more hydrogen bonds, and longer gel formation time—could not form complex coacervates, which are necessary to prepare oil powders. For oil powders obtained from the other gelatins, gelatin type and concentration did not have obvious effects on microscale morphologies; they did, however, have significant effects on physicochemical properties. The highest peroxide values of the oil powders were mainly dependent on the gelatins, expressed in the following manner: PSG (153 ± 5 – 168 ± 3 meq/Kg oil) < BSG (176 ± 5 – 188 ± 1 meq/Kg oil) < FG (196 ± 11 – 201 ± 22 meq/Kg oil). Acidic and neutral pH could not dissolve the complex coacervates. However, the oil powders could be quickly dissolved to form emulsion droplets in the gastric phase, and that SSOS increased coacervate stability and promoted oil digestion during the in vitro gastrointestinal process. In sum, this study contributes fundamental information to understanding the development of fish oil solid encapsulation preparations.

## Introduction

Gelatins have been widely developed and used in a variety of fields, including medical tissue engineering, drug delivery, cosmetics, and food science^1,2^. They can be extracted from different tissue sources (e.g., bone and skin) of different animals (e.g., mammalian, poultry, and fish)^3–5^, and, qualitatively, their functional behaviors have generally been found to be dependent on their sources^6^ and the extraction methods used to obtain them^7–10^. Therefore, it is important to investigate the effects of gelatin type on the preparation and properties of gelatin-based products.

Fish oils are rich in omega-3 polyunsaturated fatty acids and provide many significant health benefits for the health care and nutritional therapy of human beings^11,12^. Nevertheless, fish oil applications were historically limited due to the fishy odor/taste, poor water solubility, and easy oxidation^13^ of the oils. In response, fish oil encapsulation preparations have been developed to eliminate these disadvantages, leading fish oil encapsulation to become a sought-after research topic in the field of food science^14^.

There are many encapsulation methods used to develop oil preparations^15^, including complex coacervation^16,17^, spray-drying^18^, freeze-drying^19^, and electrospraying^20^. Among these, the complex coacervation method is an important and popular method that uses gelatins as wall materials for encapsulating fish oil. Previous studies have applied this method to encapsulate fish oil via the electrostatic interaction of gelatin with acacia gum^21^, sodium hexametaphosphate (SHMP)^22^, anionic gum Arabic^23^, and almond gum^24^. However, the encapsulation efficiencies of these products were generally <90%. Recently, we developed freeze-dried fish oil powders with high fish oil encapsulation efficiency (>95.2%) by combining gelatin-SHMP complex coacervation with the drying aid of starch sodium octenyl succinate (SSOS)^25^. While our research showed promising application prospects for the development and application of fish oil preparations, the detailed effect mechanisms of gelatin type and concentration on the properties of fish oil powders remain unclear.

The purpose of this study was to analyze effects of gelatin type and concentration on the preparation, physicochemical properties, and in vitro digestion behaviors of fish oil powders (named as fish oil@gelatin-SHMP@SSOS powders) using a combination method of gelatin-SHMP complex coacervation and freeze drying with aid of SSOS. Four types of gelatins were used in this work: porcine skin gelatin (PSG), bovine skin gelatin (BSG), fish gelatin (FG), and cold-water fish skin gelatin (CFG). First, the preparation process of the fish oil powders was described. Second, the effects of gelatin type and concentration on the formation of fish oil-loaded gelatin-stabilized emulsions and fish oil@gelatin-SHMP complex coacervates were studied. Third, the effects of preparation conditions on the CFG-SHMP complex coacervate formation were investigated. Fourth, the effects of gelatin type and gelatin concentration on the microscale morphologies of fish oil@gelatin-SHMP@SSOS and fish oil@gelatin-SHMP powders were analyzed. Fifth, the effects of gelatin type and concentration on the physicochemical properties of the fish oil@gelatin-SHMP@SSOS powders were determined. Sixth, the effects of gelatin type and concentration on the oil oxidative stability of the fish oil@gelatin-SHMP@SSOS powders were investigated. Seventh, the effects of gelatin type and concentration on the stability of the fish oil@gelatin-SHMP@SSOS powders at acidic and neutral pH were investigated. Finally, the effects of gelatin type and concentration on the in vitro digestion behaviors of the fish oil@gelatin-SHMP@SSOS and fish oil@gelatin-SHMP powders in simulated gastrointestinal model were analyzed.

## Results and discussion

### Preparation of the fish oil powders

**Fig. 1:**
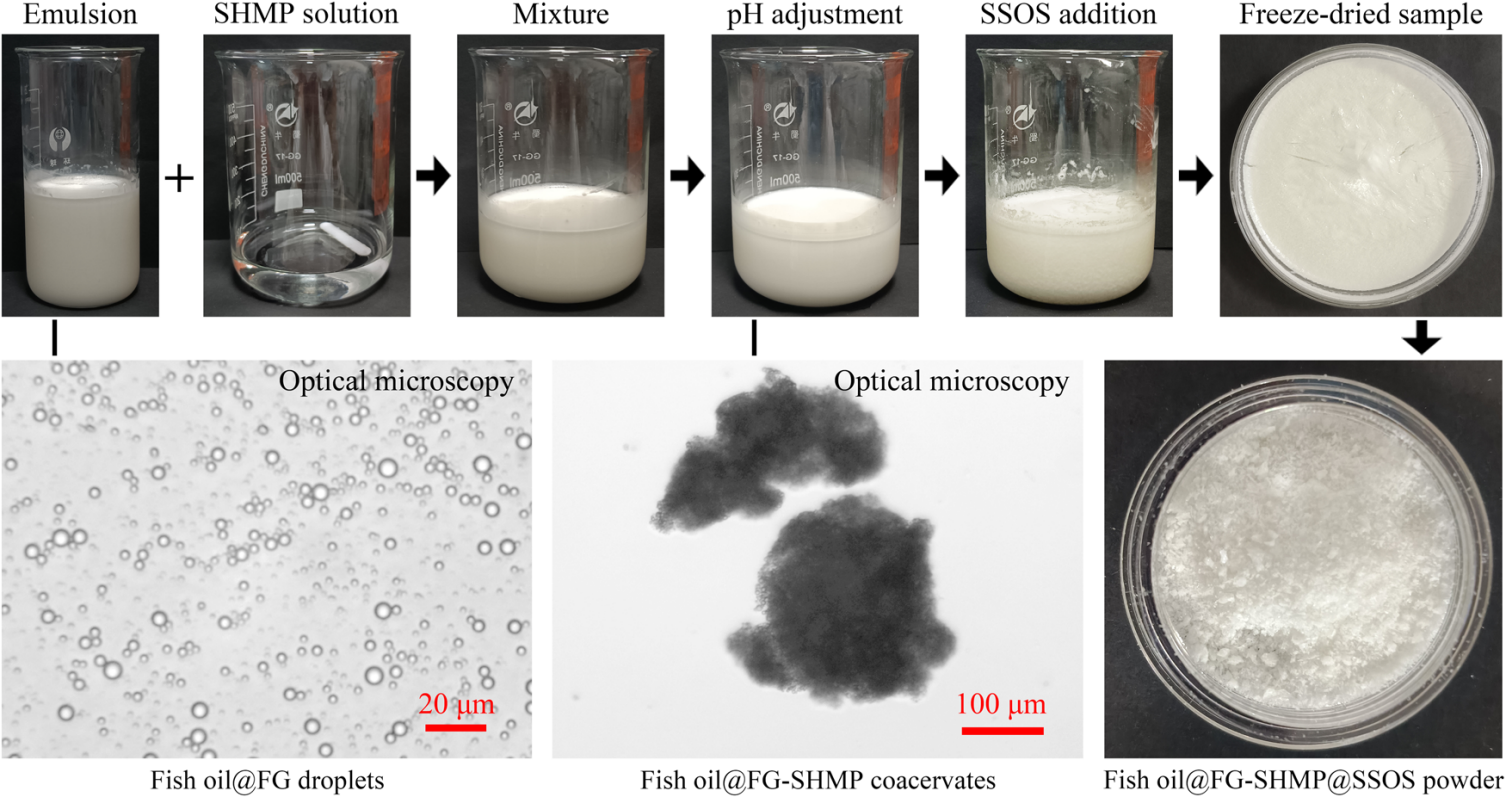
Preparation of fish oil@fish gelatin (FG)-sodium hexametaphosphate (SHMP)@starch sodium octenyl succinate (SSOS) powder. Except for the optical microscopy images, all other images were photographed using a digital camera. FG concentration was 100 mg/mL; the adjusted pH to form complex coacervates was 4.8.

A fish oil-loaded FG-stabilized emulsion was obtained using a simple homogenization method. As shown in Fig. 1, the presence of microscale (<10 μm) droplets in optical microscopy images suggested the successful preparation of a fish oil emulsion^26^. The emulsion was then mixed with SHMP solution, and pH was carefully adjusted to allow the formation of complex coacervates. As shown in the optical microscopy image in Fig. 1, microscale (>100 μm) complex coacervates appeared at pH 4.8. The gelatin molecules became positive at pH 4.8. Therefore, the electrostatic interaction between SHMP molecules and gelatin molecules occurred in the interfaces of the emulsion droplets and the gelatins in the water phase, which promoted the formation of fish oil@FG-SHMP complex coacervates. Optical microscopy was used to monitor the coacervate sizes. The pH was chosen to form appropriate coacervate sizes in the optical microscopy images: 4.7, 4.4, 4.8, and 4.7 for PSG, BSG, FG, and CFG, respectively. After SSOS dissolution, the fish oil@FG-SHMP@SSOS powders could be prepared using freeze-drying and gentle crushing (Fig. 1).

### Effects of gelatin type and concentration on the fish oil@gelatin-SHMP complex coacervate formation

**Fig. 2:**
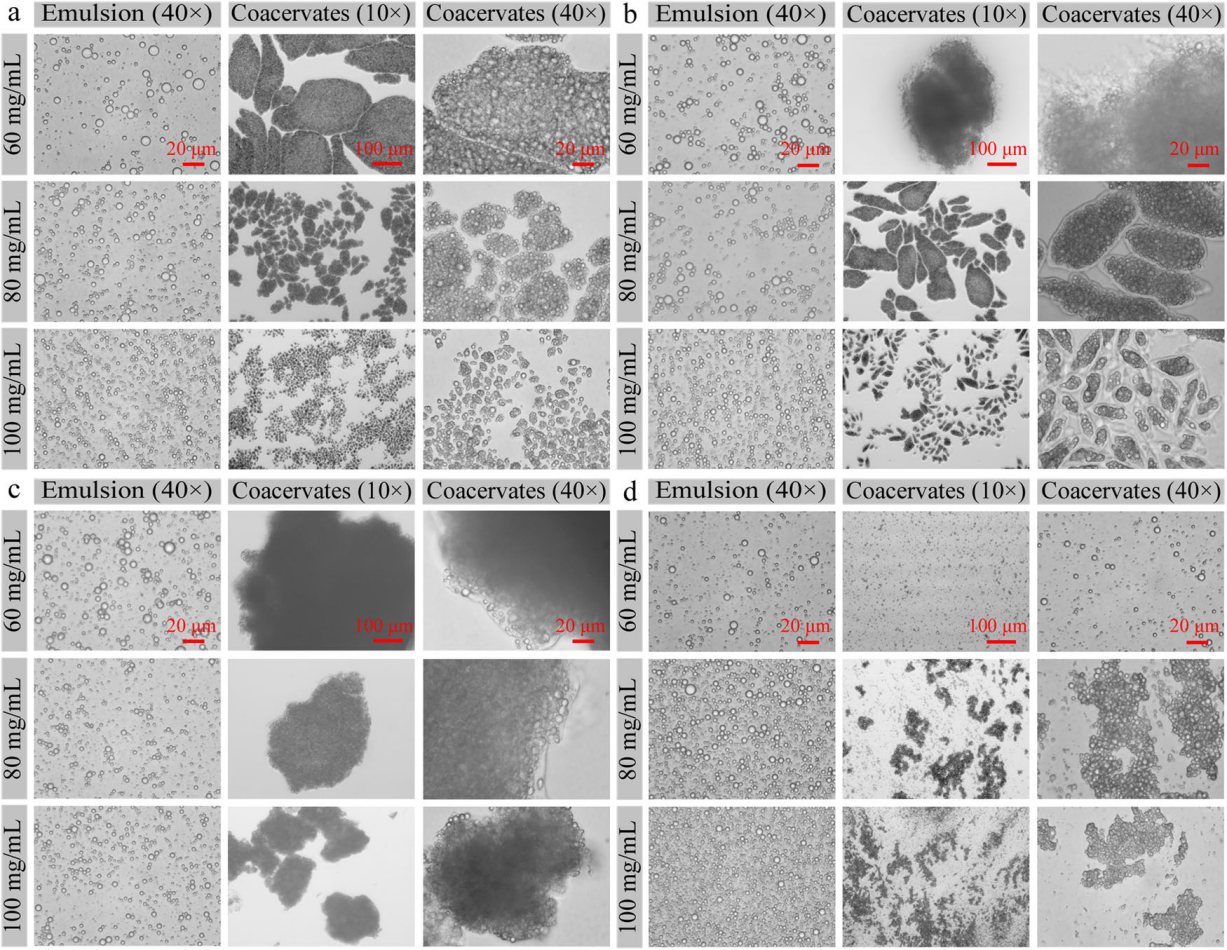
Effect of gelatin type and concentration on the preparation of fish oil-loaded gelatin emulsions and gelatin-SHMP complex coacervates. Samples were observed by an optical microscope with 10 × and 40 × objectives. **a** Porcine skin gelatin (PSG); adjusted pH to form complex coacervates was 4.7. **b** Bovine skin gelatin (BSG); adjusted pH to form complex coacervates was 4.4. **c** FG; adjusted pH to form complex coacervates was 4.8. **d** Cold-water fish skin gelatin (CFG); pH to form complex coacervates was 4.7.

The effects of gelatin type (PSG, BSG, or FG) and concentration (60, 80, or 100 mg/mL) on the preparation of the complex coacervates were investigated (Fig. 2a–c). The complex coacervates could be formed at each of the three different concentrations. According to the optical microscopy images of coacervates (10 × and 40 ×), the complex coacervate sizes decreased with the increase of the concentrations of PSG, BSG, and FG. The possible reason might be the increased “seeds” to form complex coacervates with the increase of the gelatin concentrations. As shown in Fig. 2 (coacervates 40 ×), all of the complex coacervates consisted of spherical structures. In addition, all of the emulsion sizes were significantly lower than the fish oil@gelatin-SHMP complex coacervate sizes. This suggested that all of the complex coacervates were the aggregated products of the emulsion droplets due to the electrostatic interaction between the negative SHMP molecules and the positive gelatin molecules.

### Effects of preparation conditions on the CFG-SHMP complex coacervate formation

The pH and SHMP: gelatin mass ratio are important experimental conditions to prepare complex coacervates. Previous work suggested PSG, BSG, and FG could be applied to prepare complex coacervates at pH ≤ 4.9, ≤ 4.4, and ≤ 4.8, respectively^25^. Here, fish oil@CFG-SHMP complex coacervates could be formed at pH 4.5–4.7 (Supplementary Fig. 2) and SHMP: gelatin mass ratios of 1:10–1:20 (Supplementary Fig. 3). However, these complex coacervates were floated and aggregated on the water phase to form floating layers when the magnetic stirring was stopped in 2 min (data not shown).

Gelatin concentration was another important experimental condition to prepare complex coacervates. Fish oil@CFG-SHMP complex coacervates could be only formed at gelatin concentrations of 80 and 100 mg/mL (Fig. 2d). Moreover, these coacervates were also floated and aggregated on the water phase to form floating layers when the magnetically stirring was stopped in 2 min (data not shown).

The four types of gelatins had different physicochemical properties. Type A PSG, BSG, and FG had similar isoelectric points (pIs) of 8–9^27^. CFG had no obvious pI^26^. As shown in Supplementary Fig. 1A, the molecular patterns of PSG, BSG, and FG consisted of clear or blurry bands at about 125 kDa, 140 kDa, and 280 kDa^25^. CFG had clear band of <30 kDa, which was consistent with previous SDS-PAGE result of CFG^26^. According to Supplementary Fig. 1A, the molecular weight order was: BSG > PSG > FG > CFG. Moreover, CFG had significant lower molecular weight than other gelatins.

ATR-FTIR spectra (Supplementary Fig. 2) showed that CFG had similar peak center positions of amide B (2935 cm^−1^), amide I (1636 cm^−1^), and amide III (1235 cm^−1^) to other gelatins, whereas had less peak center positions of amide A (3290 cm^−1^) and amide II (1526 cm^−1^) than other gelatins (3300 cm^−1^ of amide A and1540 cm^−1^ of amide II). It suggested, compared with other gelatins, CFG had more N-H groups that participated in the hydrogen bonds due to the lower peak position of amide A and had more hydrogen bonds between α-chains by N-H groups due to the lower peak position of amide II^28–30^. Therefore, CFG had more hydrogen bonds for molecular interaction than other gelatins, which might be due to its less molecular weight (Supplementary Fig. 1A).

The low temperature (10 °C) gel formation ability of four types of gelatins were analyzed. As shown in Supplementary Fig. 1C, only CFG solution could not form gelatin gel after 16 h at 10 °C, and therefore its gel strength (g Bloom value) could not be determined^31^. The g Bloom values of other gelatins were measured in a previous work^25^: FG (270 ± 10) < PSG (290 ± 10) < BSG (320 ± 10). Gelatin with lower molecular weight commonly required a longer time for gel formation^32^. Therefore, the low molecular weight of CFG might be the reason for that it could not form gelatin gel after 16 h at 10 °C.

Compared with other gelatins (PSG, BSG, and FG), CFG could not be used to prepare complex coacervates, even as we tried different pH values (Supplementary Fig. 2), SHMP: gelatin mass ratios (Supplementary Fig. 3), and gelatin concentrations (Fig. 2d). The possible reason might be CFG’s unobvious pI, lower molecular weight (Supplementary Fig. 1a), greater number of hydrogen bonds (Supplementary Fig. 1b), and longer gel formation time than the other three gelatins (Supplementary Fig. 1c). Moreover, the unideal complex coacervation was not dependent on the gelatin sources (FG vs. CFG), experimental pH (Supplementary Fig. 2), SHMP: gelatin mass ratio (Supplementary Fig. 3), and gelatin concentration (Fig. 2d).

### Microscale morphologies of fish oil powders

**Fig. 3:**
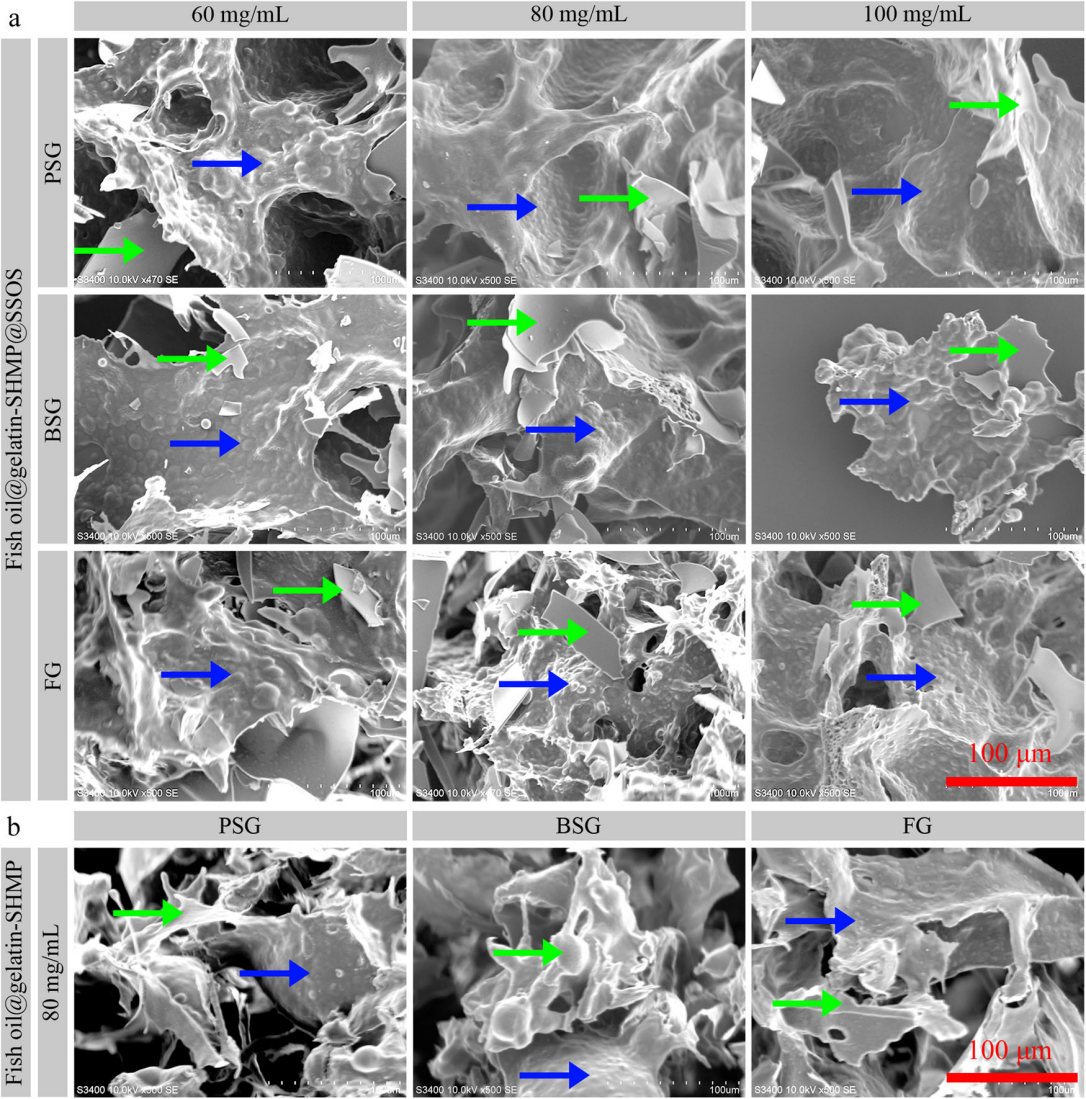
Microscale morphologies of the freeze-dried fish oil powders using scanning electron microscopy. **a** Fish oil@gelatin-SHMP@SSOS powders. **b** Fish oil@gelatin-SHMP powders. Green arrows indicate smooth surfaces; blue arrows indicate rough surfaces. All of the images have the same magnification. pH values were 4.7, 4.4, and 4.8 for PSG-, BSG-, and FG-based complex coacervates, respectively.

The microscale morphologies of the powders with or without SSOS addition were observed by SEM, as shown in Fig. 3. The images showed there were many passages for the sublimation of ice, which were the typical characteristics of freeze-dried products^33^. All of the powders consisted mainly of rough particles (micrometer-level spherical protrusions, indicated by blue arrows in Fig. 3) and minorly of smooth particles (indicated by green arrows in Fig. 3). Moreover, gelatin type and concentration had no obvious effects on the morphologies of the obtained fish oil powders.

The spherical protrusions of fish oil-loaded particles might be fish oil-wall material core-shell structures. Both the average sizes (Supplementary Fig. 4a) of the emulsion droplets in fish oil-loaded emulsions and the average sizes (Supplementary Fig. 4b) of the protrusions in SEM images were in several micrometers, which suggested that the fish oil powders with spherical protrusions might be due to the aggregation of the emulsion droplets during the gelatin-SHMP complex coacervation process^25^. According to previous works^25,34^, we could reasonably suggest that the powders consisted of two types of particles. The rough particles were fish oil-loaded particles with SSOS coating (Indicated by blue arrows in Fig. 3a) or without SSOS coating (Indicated by blue arrows in Fig. 3b). The rough surfaces were due to the presence of the emulsion droplet structures in the coacervates. The smooth particles were fish oil-unloaded particles with SSOS coating (Indicated by green arrows in Fig. 3a) or without SSOS coating (Indicated by green arrows in Fig. 3b). The smooth surfaces were due to the absence of the emulsion droplet structures in the coacervates.

SSOS addition might increase the stability of the complex coacervates during the freeze-drying process. Therefore, the spherical protrusion sizes of the fish oil@gelatin-SHMP@SSOS powders (Fig. 3a) were larger than those of fish oil@gelatin-SHMP powders (Fig. 3b), which is consistent with the fact that starches were good drying aid choices for emulsion freeze-drying^35^.

### Effects of gelatin type and concentration on the physicochemical properties of the fish oil powders

**Fig. 4:**
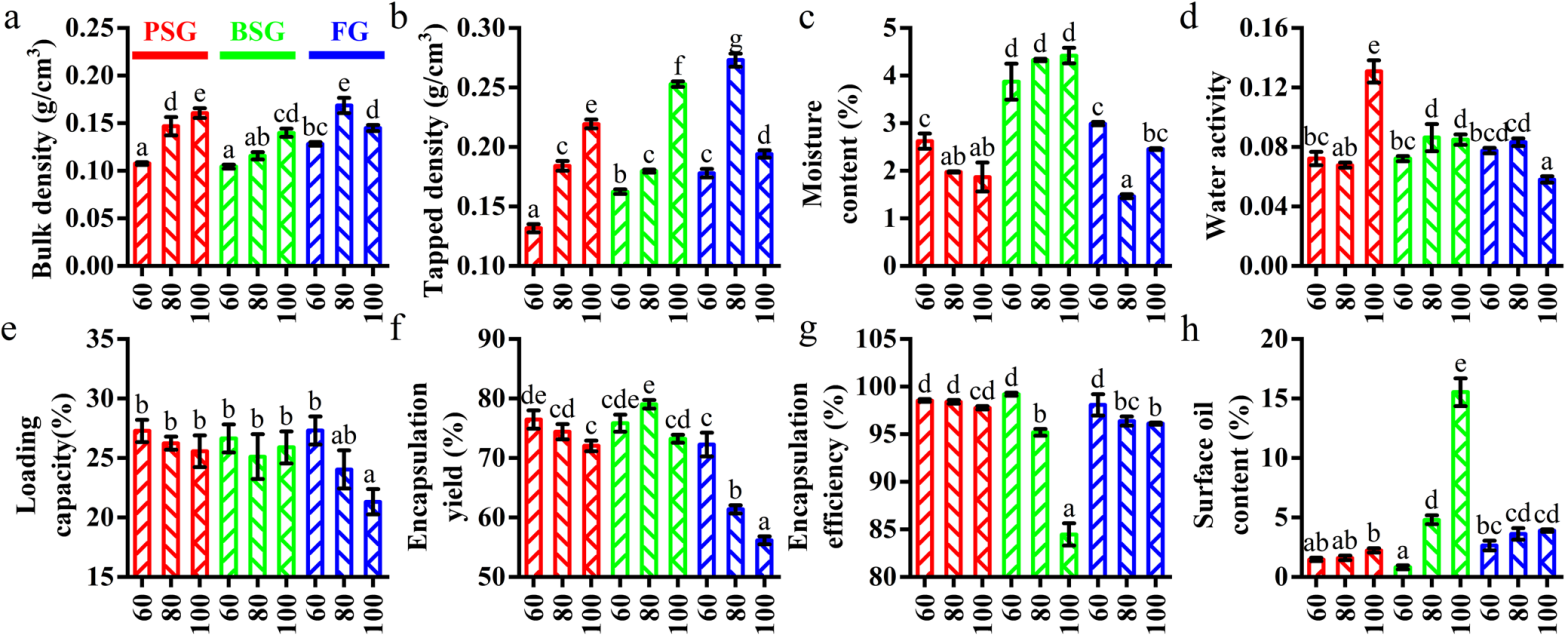
Effect of gelatin type and concentration on the physicochemical properties of the fish oil@gelatin-SHMP@SSOS powders. **a** Bulk density. **b** Tapped density. **c** Moisture content. **d** water activity. **e** Loading capacity. **f** Encapsulation yield. **g** Encapsulation efficiency. **h** Surface oil content. Error bars refer to standard deviation of the mean. Significant (*p* < 0.05) differences are indicated by different lowercase letters.

The effects of gelatin type (PSG, BSG, and FG) and concentration (60, 80, and 100 mg/mL) on the common physicochemical parameters of the fish oil@gelatin-SHMP@SSOS powders were analyzed, as shown in Fig. 4. The results showed that these parameters were dependent on both gelatin type (animal and tissue sources) and concentration.

Research has demonstrated that bulk and tapped densities can be used to reflect the particle sizes of a powder sample, where the values decreased with the increase of particle size for a powder sample^36^. According to previous work^37^, bulk and tapped densities (g/cm^3^) could be affected by the wall material composition. For the powders in this study (Fig. 4a, b), the bulk and tapped densities were dependent on gelatin type (animal and tissue sources). Specifically, both PSG and BSG induced increased densities (decreased particle sizes) with the increase of gelatin concentration, whereas FG did not. Among these powders, the powder at a gelatin concentration of 80 mg/mL had the highest density.

The moisture content and water activity of the powders were analyzed (Fig. 4c, d). Both elements were found to be dependent on gelatin type (animal and tissue sources) and gelatin concentration. Among the powders in this study, fish oil@BSG-SHMP@SSOS powders had the highest moisture content. Moreover, the powder at a PSG concentration of 100 mg/mL had the highest water activity. It might result from that the difference of PSG to other gelatins and the high PSG concentration (100 mg/mL). The detailed mechanism should be further studied in the future.

Fish oil LC and EY are key parameters to producing oil-encapsulated food products^38^. For the powders in this study (Fig. 4e, f), values were dependent on gelatin type (animal and tissue sources) and concentration. As shown in Fig. 4e, gelatin concentration had no significant effect on the LC values of PSG- and BSG-based powders. However, the LC values of FG-based powders decreased with the increase in gelatin concentration. It might result from the composition and structural difference of aquatic FG to mammalian gelatins (PSG and BSG)^27^. As shown in Fig. 4f, the EY values of PSG-based and FG powders decreased with the increase in gelatin concentration, whereas BSG-based powders showed the highest value (79.01%) at the gelatin concentration of 80 mg/mL. The EY values confirmed that fish oil was not encapsulated in or on the fish oil powders, and they also confirmed the presence of many passages for the sublimation of ice in the SEM images (Fig. 3).

Fish oil EE and surface oil content are additional key parameters to producing oil-encapsulated food products^38^. For the powders in this study (Fig. 4g, h), values were dependent on gelatin type (animal and tissue sources) and concentration. All of the powders had high fish oil EE values of >95% and low surface oil contents of < 5%, except the BSG-based powder at a gelatin concentration of 100 mg/mL (Fig. 4g, h). The EE values of most of the powders were higher than that (88.03%) of tuna oil@gelatin-SHMP powder^22^. With the increase of gelatin concentrations (60–100 mg/mL), EE values decreased and surface oil content values increased. Moreover, the trend was dependent on gelatin type, where BSG > FG > PSG. Therefore, BSG-based powders at a BSG concentration showed the lowest EE value among these powders. The EE values of thyme essential oil-loaded β-cyclodextrin/gum Arabic microcapsules increased and then decreased with the increase of gum Arabic content^39^. Therefore, it was necessary to explore more gelatin concentrations to provide a complete representation of the effect of gelatin concentrations on the EE and surface oil content values of gelatin-based powders. As the pulverized powder becomes smaller, the entrapped oil is exposed, and the surface oil increases. At the increasing gelatin concentrations of PSG- and BSG-based powders, the EE values showed trends that were contrary to the bulk and tapped densities (Fig. 4a, b), indicating that the EE values showed trends that were similar to the particle sizes of the PSG- and BSG-based powders. However, the FG-based powders did not show relationships between EE values and particle sizes similar to PSG- and BSG-based powders (Fig. 4a, b, g). Therefore, particle size was not a key parameter for affecting the EE and surface oil content values of the powders.

It was interesting that BSG (100 mg/mL) induced the lowest EE and the highest surface oil contents among these samples. At similar loading capacity (Fig. 4e) and encapsulation yield (Fig. 4f), the BSG (100 mg/mL) induced the lowest encapsulation efficiency (Fig. 4g) and the highest surface oil content (Fig. 4g). It suggested fish oil was not well encapsulated in the powder particle core at this condition. High BSG concentration (100 mg/mL) might be the main reason for it. The detailed mechanism should be further studied in the future.

### Oxidative stability of fish oil powders

During the preservation process, oil preparations generally showed increased oxidation with time. After oxidation, primary lipid hydroperoxides were formed and then converted into secondary oxidation products such as volatile compounds, malonaldehyde, and anisidine^40^. Under a Schaal oven test condition, the storage of oil preparations at 63 °C for 1 day was equivalent to 16 days at room temperature^41,42^.

**Fig. 5:**
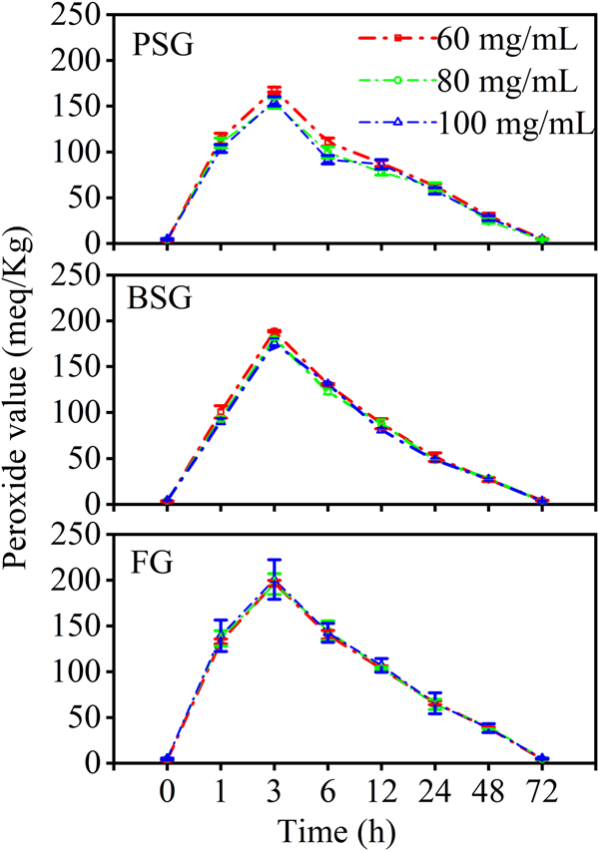
Peroxide values of the fish oil@gelatin-SHMP@SSOS powders (63 °C, 72 h). Error bars refer to standard deviation of the mean.

Under a Schaal oven test condition, the primary lipid hydroperoxides of the fish oil powders were analyzed by measuring peroxide values (Fig. 5). For all of the powders, the peroxide values increased with time at the initial 3 h and then decreased. This trend was consistent with the peroxide value change trends of fish oil-loaded calcium alginate and calcium alginate/Span capsules^43^. Moreover, the conversion time point of 3 h was the same as the fish oil-loaded calcium alginate capsules^43^. Therefore, during the Schaal oven test condition, the primary lipid hydroperoxides were formed and then converted to secondary oxidation products^44^.

Peroxide values were not dependent on gelatin concentrations but rather on gelatin types. At 3 h, the peroxide values of the fish oil powders were mainly dependent on the gelatins, expressed in the following manner: PSG (153 ± 5 – 168 ± 3 meq/Kg oil) < BSG (176 ± 5 – 188 ± 1 meq/Kg oil) < FG (196 ± 11 – 201 ± 22 meq/Kg oil); this suggested that gelatins have different preservation abilities for fish oil powders, where PSG < BSG < FG. The values were lower than the peroxide value (308 ± 12 meq/Kg oil) of fish oil-loaded calcium alginate capsules at 3 h^43^ and similar to that (about 210 meq/Kg oil) of fish oil-loaded calcium alginate capsules at 17 days after 37 °C incubation^45^. These results implied that the freeze-dried gelatin powder preparation might be better than calcium alginate capsules for the storage of fish oil. At the increasing gelatin concentrations, the trends of the peroxide values did not show obvious relationships to the bulk and tapped densities (Fig. 4a, b). Therefore, the oxidative stability of fish oil powders did not show obvious relationships to the particle sizes of the powders.

### Stability of the fish oil powders at acidic and neutral pH

**Fig. 6:**
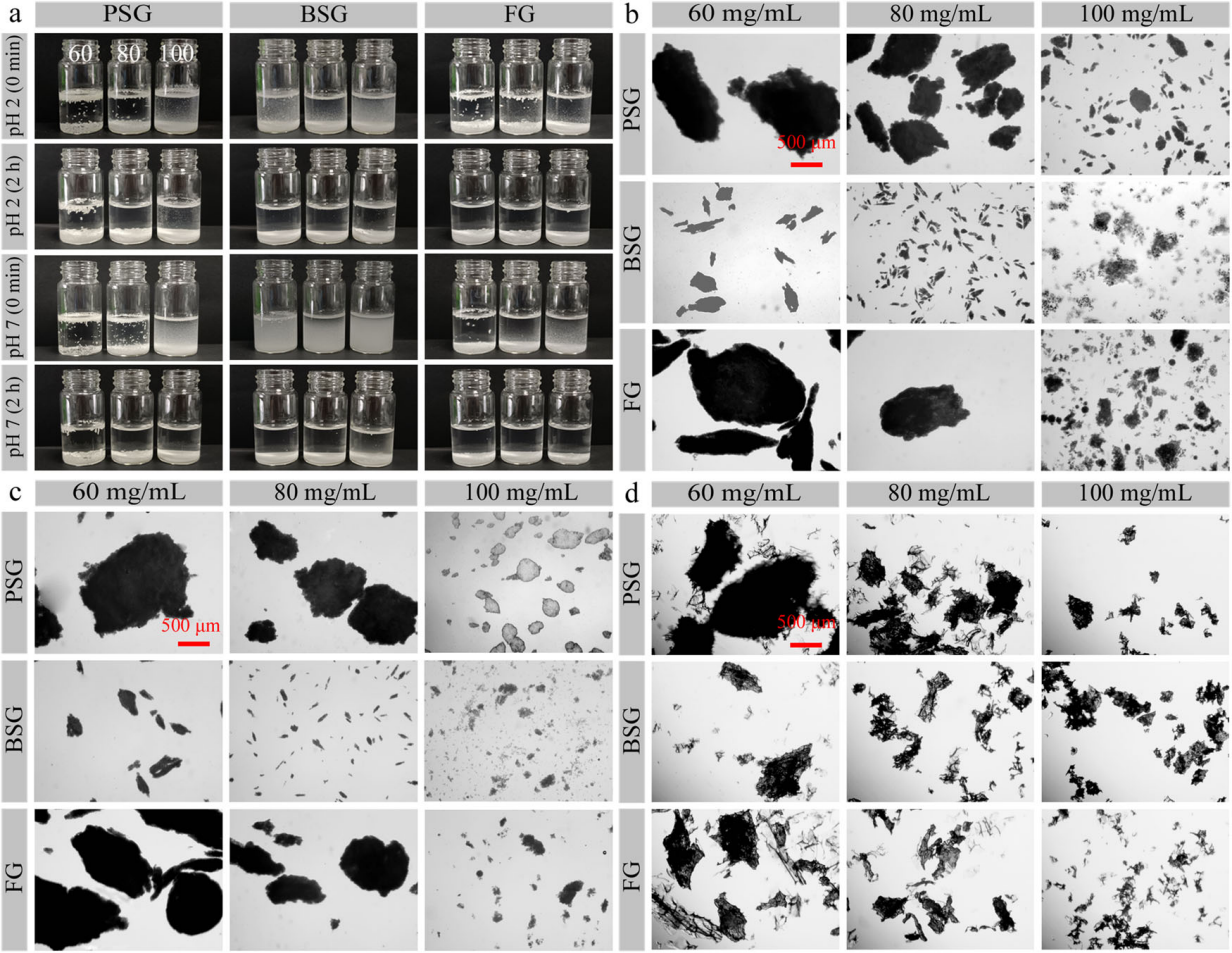
Observation of the powders with SSOS addition at different gelatin concentrations (60, 80, and 100 mg/mL) during incubation in phosphate-buffered saline (PBS) solutions (pH 2.0 or 7.0) at room temperature. **a** Digital camera images of the powders in PBS at different incubation times (0 min and 2 h). Gelatin concentrations from left to right are 60, 80, and 100 mg/mL in each image. **b** Optical microscopy images of the powders after 2-h incubations in PBS (pH 2.0). **c** Optical microscopy images of the powders after the 2–h incubation in PBS (pH 7.0). **d** Optical microscopy images of the untreated powders. All of the images have the same magnification.

PBS solutions at pH 2.0 and 7.0 were, respectively, simple gastric and small intestinal models used to investigate the possible in vitro digestion of the core-shell fish oil-loaded Ca^2+^-alginate capsules^20^. Here, the powders were put into PBS solutions (pH 2.0 and 7.0) and were observed using digital camera techniques and optical microscopy (Fig. 5 and Supplemenatry Figs. 5, 6). Even after 2-h incubation, there were still many precipitates in the glass vials (Fig. 6a). The precipitates (Fig. 6b, c) after 2-h incubation consisted of particles, which were similar to the untreated powders (Fig. 6d). Considering the fact that SSOS is water-soluble, the SSOS layers of the powders might be dissolved in the PBS, and the precipitates present might be the obtained complex coacervates that were formed at an acidic pH (Fig. 2). Therefore, acidic and neutral PBS were not appropriate models to analyze the potential digestion behaviors of the powders. It also demonstrated the acidic and neutral pH could not dissolve the gelatin-SHMP complex coacervates.

### In vitro digestion mechanism of the fish oil powders

**Fig. 7:**
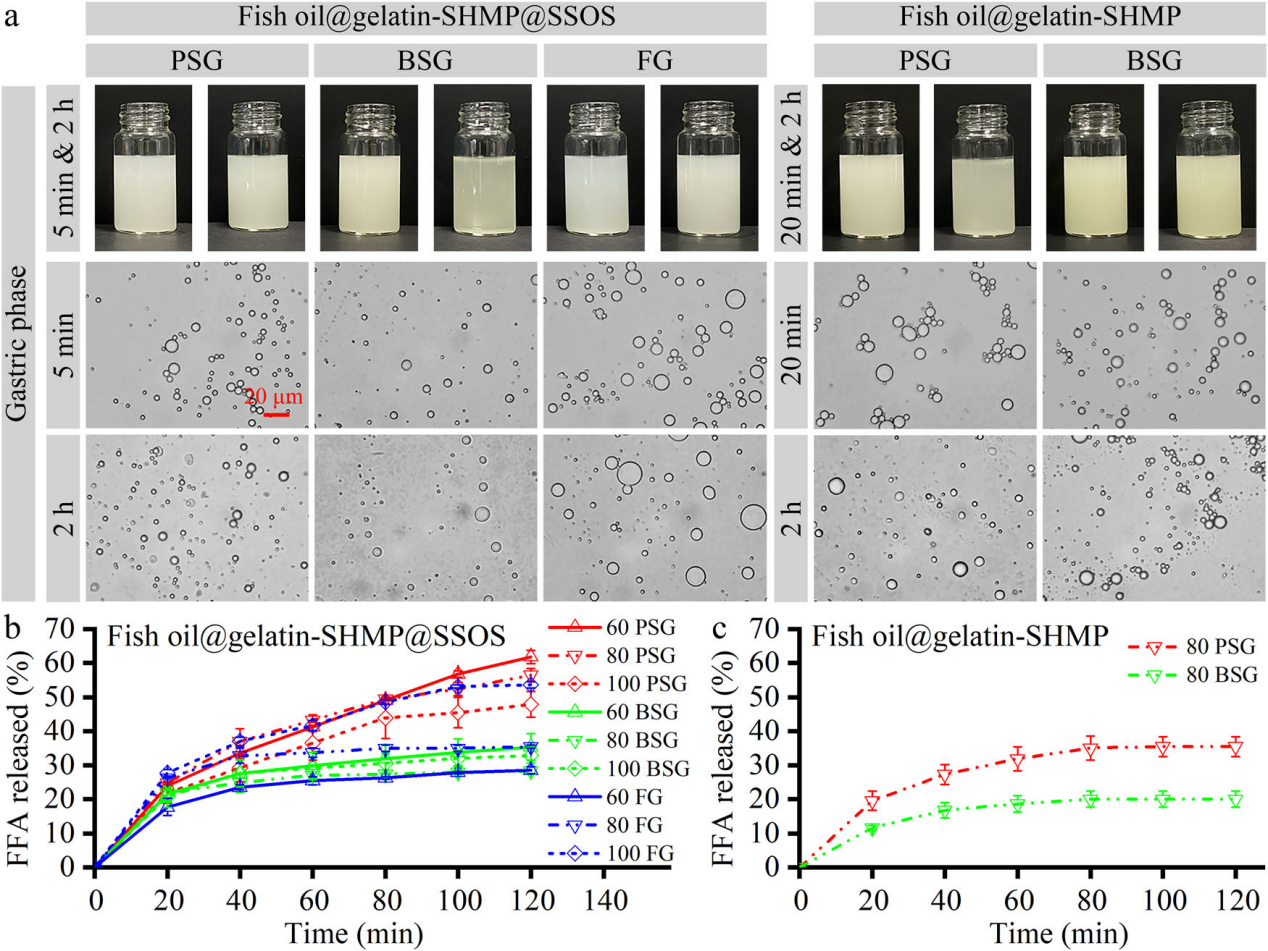
In vitro digestion behaviors of the powders with or without SSOS addition in the simulated gastrointestinal model at room temperature. **a** Digital camera and optical microscopy observations of the powders at a gelatin concentration of 80 mg/mL in the gastric phase. All of the optical microscopy images have the same magnification. **b** Free fatty acid (FFA) released percentages of the powders with SSOS addition at different gelatin concentrations (60, 80, and 100 mg/mL) in the small intestinal phase. **c** FFA released percentages of fish oil@gelatin-SHMP powders at a gelatin concentration of 80 mg/mL in the small intestinal phase. Error bars refer to standard deviation of the mean.

The digestion behaviors of the powders with or without SSOS addition were studied using an in vitro gastrointestinal system^25,46^. These powders, at a gelatin concentration of 80 mg/mL in simulated gastric phase, were examined using digital camera techniques and optical microscopy (Fig. 7a). In addition, the powders at different gelatin concentrations (60, 80, and 100 mg/mL) after the in vitro gastrointestinal system were also examined in Supplemenatry Fig. 7. Finally, the FFA released percentages (Fig. 7b, c) of the fish oils in the small intestinal phase were also analyzed.

In the gastric phase (Fig. 7a: First line), the fish oil@gelatin-SHMP@SSOS powders and the fish oil@gelatin-SHMP powders disappeared at 5 and 20 min, respectively, in the glass vials (shown in the digital camera images), which were different to the undissolved precipitates in the glass vials at the acidic and neutral pH PBS (Fig. 6a). The optical microscopy images (Fig. 7a: Second and third lines) showed the presence of the emulsion droplets, which were different to the undissolved precipitates at the acidic and neutral pH PBS (Fig. 6b–d). Therefore, all of the powders with or without SSOS were quickly dissolved (shown in the digital camera images) to form emulsion droplets (shown in the optical microscopy images). It suggested that the complex coacervates could change into emulsion droplets after SSOS dissolution. Therefore, the in vitro gastrointestinal system might be a better model than the PBS solution (Fig. 6) to study the digestion of the fish oil powders.

After the small intestinal phase (Supplemenatry Fig. 7), the emulsion droplet amounts of all of the samples at different concentrations (60, 80, and 100 mg/mL) were decreased compared with those in the gastric phase (shown in the optical microscopy images). A previous study also showed the presence of emulsion droplets of fish oil@gelatin-SHMP@SSOS samples at a gelatin concentration of 80 mg/mL after gastrointestinal incubation^25^. FFA released percentages of the samples with (Fig. 7b) and without (Fig. 7c) SSOS addition confirmed the incomplete digestion behaviors of fish oil, which also implied that the emulsion droplets were not completely destroyed in the small intestinal phase.

SSOS showed an obvious effect on the in vitro digestion of the powders. In the gastric phase (Fig. 7a), the powders with SSOS addition were dissolved in 5 min, whereas the powders without SSOS addition were dissolved in 20 min. In the small intestinal phase, the FFA released percentages (Fig. 7b) of the samples with SSOS addition were significantly higher than those (Fig. 7c) of the samples without SSOS addition. Therefore, SSOS promoted the transformation of the complex coacervates to emulsion droplets in the gastric phase and the destruction of the emulsion droplets in the small intestinal phase, indicating that SSOS promoted the digestion of fish oil in the powders.

Gelatin type and concentration displayed obvious effects on the FFA release behaviors of the fish oil@gelatin-SHMP@SSOS powders. For the PSG-based samples, the FFA released percentages decreased with the increase in gelatin concentration. For the BSG-based samples, the sample at a gelatin concentration of 60 mg/mL had the highest FFA released percentages. For the FG-based samples, the FFA released percentages increased with the increase in gelatin concentration. These trends did not show obvious consistency to the physicochemical properties of the powders (Fig. 4). However, it might result from the differences of the gelatin types and concentrations. The detailed mechanisms should be further studied in the future. Therefore, it is necessary to carefully explore the effects of the gelatin type and concentration for the ideal preparation of gelatin-based powders in the development of fish oil preparations.

In this study, the effect of gelatin type and concentration on the preparation and properties of fish oil@gelatin-SHMP@SSOS powders were comprehensively studied. Results suggested that not all of the gelatins could be applied in the preparation of the powders. CFG—with a lower molecular weight, more hydrogen bonds, and longer gel formation time—could not be used to prepare complex coacervates and fish oil powders. In addition, gelatin type and concentration had significantly different effects on the powders. Under a Schaal oven test condition, the highest peroxide values of the fish oil powders were mainly dependent on the gelatins, expressed in the following manner: PSG (153 ± 5 – 168 ± 3 meq/Kg oil) <BSG (176 ± 5 – 188 ± 1 meq/Kg oil) <FG (196 ± 11 – 201 ± 22 meq/Kg oil). This work was beneficial for the development of solid encapsulation preparations of fish oils, and it could also provide useful fundamental information for understanding the effect of gelatin-based wall materials on the preparation and properties of fish oil solid encapsulation preparations. Further work is necessary to explore the effects of gelatin tissue sources, animal sources, and extraction methods on the powders, which would be beneficial for cultivating a basic understanding of the protein structure-function relationship and the high-value utilization of agricultural by-products. In addition, it is necessary to explore the effect of particle sizes on the properties of the powders by preparing the powders with the same materials and the same preparation concentrations. Finally, it is also interesting to analyze the complex coacervation in the gelatin-SHMP system without fish oil, which might increase our understanding on the complex coacervation formation process.

## Methods

### Emulsion preparation

Fish oil-loaded emulsions stabilized by different gelatins were prepared using a simple homogenization method^47^. PSG (Type A) and CFG were purchased from Sigma-Aldrich, Shanghai, China. BSG (Type A) and FG (Type A, from tilapia) were purchased from Shanghai Yuanye Bio-Technology, Shanghai, China. Briefly, 6, 8, or 10 g of gelatin was added into 100 mL of ultrapure water (yielding concentrations of 60, 80, and 100 mg/mL, respectively). Concentrations were chosen according to the sizes of coacervates obtained in optical microscopy images. Preparations were allowed to stand for 30 min and were then heated at 45 °C for 1 h to dissolve the gelatin. After cooling down to room temperature, 8 g of fish oil was added, and the mixtures were mechanically homogenized for 5 min at a speed of 8000 rpm using a T25 ULTRA-TURRAX® machine. The emulsion droplets were then observed using an ML-8000 upright optical microscope (Shanghai Minz Precision Instruments Co. Ltd., Shanghai, China).

### Complex coacervation process

The gelatin-SHMP complex coacervates were prepared using a simple pH adjustment method^22,25^. The obtained emulsions (Section 2.1) were mixed with 100 mL of SHMP solution at a stirring speed of 400 rpm at 50 °C for 1 min. The mass ratio of SHMP to gelatin was 1:15. Then, the pH was carefully adjusted by 1 mol/L of phosphoric acid solution and 1 mol/L of NaOH solution to form complex coacervates at 50 °C. Samples were stewed at room temperature for further study.

### Freeze-drying process

After the complex coacervation process (Section 2.2), 8 g of SSOS was added and the mixture was stirred at a speed of 400 rpm for 30 min. The mixture was freeze-dried at -50 °C in a lyophilizer (FD-1C-50, Beijing Boyikang Experimental Equipment Co., Ltd., Beijing, China) for 48 h. The obtained sample was gently crushed by hand to obtain fish oil powders^25^. The crushed powders were observed using a Hitachi S-3400 scanning electron microscope (SEM, Tokyo, Japan) with an accelerating voltage of 10.0 kV^48^.

### Sodium dodecyl sulfate-polyacrylamide gel electrophoresis (SDS-PAGE)

According to our previous work, the molecular weight (MW) distribution of gelatins were analyzed using DYY-6D electrophoresis apparatus (Beijing Liuyi Instrument, Beijing, China)^49^. Briefly, 2 mg/mL of gelatin at pH 7.0 was mixed with 5× SDS-PAGE sample loading buffer (GBCBIO Technologies Inc., Guangzhou City, Guangdong Province, China) at a volume ratio of 1:4 and boiled for 5 min. Then, 10 μL of the sample was loaded into 8% SurePAGE Bis-Tris gel (GenScript, Nanjing City, Jiangsu Province, China). The gel with treated with a voltage of 120 V for 80 min. Subsequently, the gel was stained for 3 h using a mixture of 0.1% (w/v) Coomassie Brilliant Blue R-250, 25% (v/v) isopropanol, and 10% (v/v) acetic acid. Finally, the gel was destained for 12 h using a mixture of 20% (v/v) ethanol and 10% (v/v) acetic acid. The destained gel was photographed using a digital camera.

### Attenuated total reflectance-Fourier transform infrared (ATR-FTIR) spectrometry

The ATR-FTIR structural characteristics of gelatins were analyzed by a PerkinElmer Spotlight 400 ATR-FTIR spectrometer (Waltham, Massachusetts, USA)^48^. The wavenumber range was 400–600 cm^−1^. The scanning resolution was 8 cm^−1^. The accumulation scan number was 32.

### Preparation of gelatin gel for gel strength measurement

The gelatin gels were prepared in glass Bloom bottles according to the British Standard 757:1975 method^31,50^. Briefly, 105 mL of ultrapure water and 7.5 g of gelatin were mixed and incubated (65 °C) for 10 min to dissolve the gelatin. The solution was left at 10 °C for 16 h to form gelatin gels.

### Bulk and tapped densities

The powders were added into a tared graduated cylinder (50 mL), and the powder volume was recorded. Then, the graduated cylinder was repeatedly tapped manually until no obvious volume change was observed, and the tapped powder volume was recorded. The bulk and tapped densities (g/cm^3^) were calculated by dividing the fish oil powder weight by the powder volume and tapped powder volume, respectively^25^.

### Moisture content and water activity

The moisture content of the powders was calculated by dividing the moisture weight differences between the initial powder weights and the constant powder weights after heating at 105 °C (2 h heating, weighing, 0.5 h heating, weighing, 0.5 h heating, and weighing; the mass difference between last two weighings was less than 2 mg) by the initial powder weights and multiplying by 100^25,51^. The water activity of the powders was measured by a professional water activity meter (AquaLab 4TE, Meter Group, Pullman, WA, USA).

### Loading capacity, encapsulation efficiency, surface oil content, and encapsulation yield

The surface oil masses of the fish oil powders were determined using a dissolution-evaporation-weighing method^52^. The powders were mixed with 10 mL of hexane and then filtered through a Whatman No. 1 filter paper. Subsequently, 10 mL of hexane was used to wash the tubes and filtered into the mixtures. The hexane washing process was repeated twice, and then the filter papers were washed with 10 mL of hexane. All of the hexane solutions were mixed, and the mixtures were rotary evaporated. Finally, the samples were heated to a constant weight (surface oil mass of the powder) at 105 °C.

Total oil (both surface oil and encapsulated oil) masses of the fish oil powders were determined using a disruption-dissolution-evaporation-weighing method^25^. The powders were disrupted into a mixture of HCl (4 mol/L, 10 mL) and hexane (20 mL). The mixtures were magnetically stirred for 8 h and kept for 30 min without stirring. The supernatants were filtered through a Whatman No. 1 filter paper. Next, 20 mL of hexane was added to the powders. Mixtures were then magnetically stirred for 30 min and the supernatants were filtered. This hexane-washing process was repeated twice, and then the filter paper was washed with 10 mL of hexane. All of the hexane solutions were mixed, and the mixtures were rotary evaporated. Finally, the samples were heated to a constant weight (total oil mass of the powder) at 105 °C.

Loading capacity (LC, %), EE (%), surface oil content (%), and encapsulation yield (EY, %) of the fish oil powders were calculated according to the following equations:

1 $${Loading}\,{capacity}\,( \% )=\frac{{Total}\,{oil}\,{mass}}{0.500}\times 100$$

2 $${Encapsulation}\,{efficiency}\,( \% )=\frac{{Total}\,{oil}\,{mass}-{Surface}\,{oil}\,{mass}}{{Total}\,{oil}\,{mass}}\times 100$$

3 $${Surface}\,{oil}\,{content}\,( \% )=\frac{{Surface}\,{oil}\,{mass}}{{Total}\,{oil}\,{mass}}\times 100$$

4 $${Encapsulation}\,{yield}\,( \% )=\frac{{Total}\,{oil}\,{mass}}{{Used}\,{oil}\,{for}\,{oil}\,{powder}\,{preparation}}\times 100$$

### Fish oil oxidative stability evaluation

The fish oil oxidative stability of the fish oil powders (0.5 g) was evaluated under a Schaal oven test condition and according to the Chinese National Standard 5009.227-2016, “Determination of Peroxide Values in Food”^43^. The powders were put in 20-mL glass vials, and samples (without sealing) were incubated at 63 °C and 70% relative humidity in an oven. Then, 30 mL of acetic acid/isooctane with a volume ratio of 3:2 was pipetted into the glass vials. After vibrating for 5 s and stewing for 10 min, 1 mL of saturated potassium iodide solution was added. After vibrating for 30 s and stewing for 3 in dark conditions, 100 mL was put into the glass vials. Then, 0.001 mol/L of sodium thiosulfate solution was added in a dropwise manner until the yellow iodine color almost disappeared. Subsequently, 1 mL of starch indicator solution (10 g/L) was added, and the mixture was vibrated until the blue color disappeared. Blank sample titration was achieved and used as the control. The peroxide value of the fish oil powders was calculated according to the following equation and expressed as milliequivalent (meq) per Kg of oil:

5 $${\rm{Peroxide\; value}}=1000\times \frac{({V}_{3}-{V}_{4})c}{{m}_{5}{LC}}$$

where *V*_3_ and *V*_4_ are the titrated volumes (mL) of sodium thiosulfate solution for the fish oil powders and blank sample, respectively; *c* is the concentration (0.001 mol/L) of sodium thiosulfate solution; *m*_5_ is the weight (g) of the used capsule; and *LC* is fish oil loading capacity (%).

### Stability at acidic and neutral pH

The fish oil powders (0.1 g) were put into 10 mL of acidic phosphate-buffered saline (PBS) solution (NaH_2_PO_4_, 10 mmol/L; pH 2.0, adjusted with phosphoric acid) or neutral PBS solution (NaH_2_PO_4_, 8.4 mmol/L; Na_2_HPO_4_, 1.6 mmol/L; pH 7.0). The mixtures were incubated for 2 h at room temperature^20^. The fish oil powder suspensions were observed by a digital camera at 0 and 120 min. The fish oil powders in the suspensions were observed by an ML-8000 upright optical microscope (Shanghai Minz Precision Instruments).

### In vitro digestion experiments

The digestion behaviors of powders were analyzed using a simulated gastrointestinal model with gastric and small intestinal phases^25,46^. Briefly, 0.1 g of fish oil powder was put in the gastric phase at 37 °C, and a pH of 2.0 was maintained for 2 h. Subsequently, the mixtures were treated in the intestinal phase at 37 °C for 2 h, and pH was maintained using 0.5 mol/L NaOH. The fatty acid (FFA) released percentages were calculated based on the following equation:

6 $${FFA}\,{released}\,\left( \% \right)=\frac{\left ({NaOH}\,{volume}\times {NaOH}\,{molarity}\times {fish}\,{oil}\,{molecular}\,{weight}\right)}{{Powder}\,{mass}\times {LC}\times {FFA}\,{molecules}\,{produced}\,{per}\,{triacylglycerol}}\times 100$$

where the fish oil molecular weight is 868 g/mol, *LC* is the fish oil loading capacity in Section 2.6, and the FFA molecules produced per triacylglycerol is 2.

### Statistical analysis

Experimental data were expressed as mean ± standard deviation from three parallel experiments. Statistical comparisons to analyze differences were carried out using one-way ANOVA; a *p*-value < 0.05 was considered statistically significant.

### Reporting summary

Further information on research design is available in the Nature Research Reporting Summary linked to this article.

### Supplementary information

Supplementary Material

Reporting Summary

## Data Availability

Data is available on request.
