# Supplementary material for: Effects of gelatin type and concentration on the preparation and properties of freeze-dried fish oil powders
Source: NPJ Sci Food. 2024 Feb 3;8:9. doi: 10.1038/s41538-024-00251-4 (PMC10837155; doi:10.1038/s41538-024-00251-4)
Supplement: Supplementary file 1 — Supplementary Material [file 41538_2024_251_MOESM1_ESM.pdf]

Supplementary Materials

Effects of gelatin type and concentration on the preparation and properties of freeze-dried fish oil powders

Mengyang Yang[1,2,†], Jiawei Peng[1,2,†], Cuiping Shi[1], Ye Zi[2], Yulu Zheng[2], Xichang Wang[2], Jian Zhong[1,2,3,4,*]

[1]Shanghai Key Laboratory of Pediatric Gastroenterology and Nutrition, Shanghai Institute for Pediatric Research, Xinhua Hospital, Shanghai Jiao Tong University School of Medicine, Shanghai 200092, China

[2]National R&D Branch Center for Freshwater Aquatic Products Processing Technology (Shanghai), Integrated Scientific Research Base on Comprehensive Utilization Technology for By-Products of Aquatic Product Processing, Ministry of Agriculture and Rural Affairs of the People's Republic of China, Shanghai Engineering Research Center of Aquatic-Product Processing and Preservation, College of Food Science & Technology, Shanghai Ocean University, Shanghai 201306, China

[3]Department of Clinical Nutrition, College of Health Science and Technology, Shanghai Jiao Tong University School of Medicine, Shanghai 200135, China

[4]Marine Biomedical Science and Technology Innovation Platform of Lingang Special Area, Shanghai 201306, China

[†]These authors contributed equally.

[*]Corresponding author at: Shanghai Key Laboratory of Pediatric Gastroenterology and Nutrition, Shanghai Institute for Pediatric Research, Xinhua Hospital, Shanghai Jiao Tong University School of Medicine, Shanghai 200092, China

E-mail: jzhong@shsmu.edu.cn (J. Zhong)

Abbreviated running title: Effects of gelatin type and concentration on oil powders

Supplementary Figures:

Supplementary Figure 1.

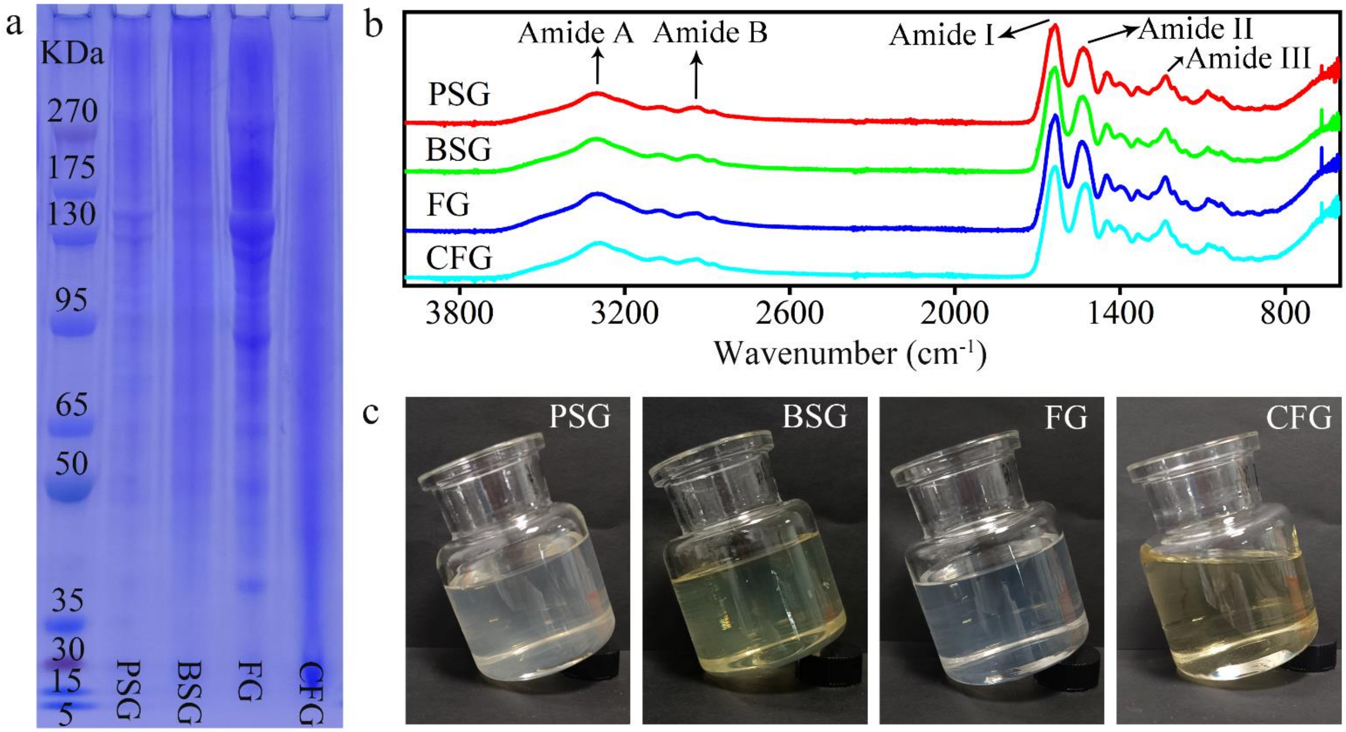

Supplementary Figure 1. Physicochemical properties of four types of gelatins: Porcine skin gelatin (PSG), bovine skin gelatin (BSG), fish gelatin (FG), and cold-water fish skin gelatin (CFG). (A): SDS-PAGE result. The left band is protein standard. (B): ATR-FTIR spectra. (C): The gelatin samples in glass Bloom bottles. The gel strength of CFG was not measured because it could not form gel.

Supplementary Figure 2.

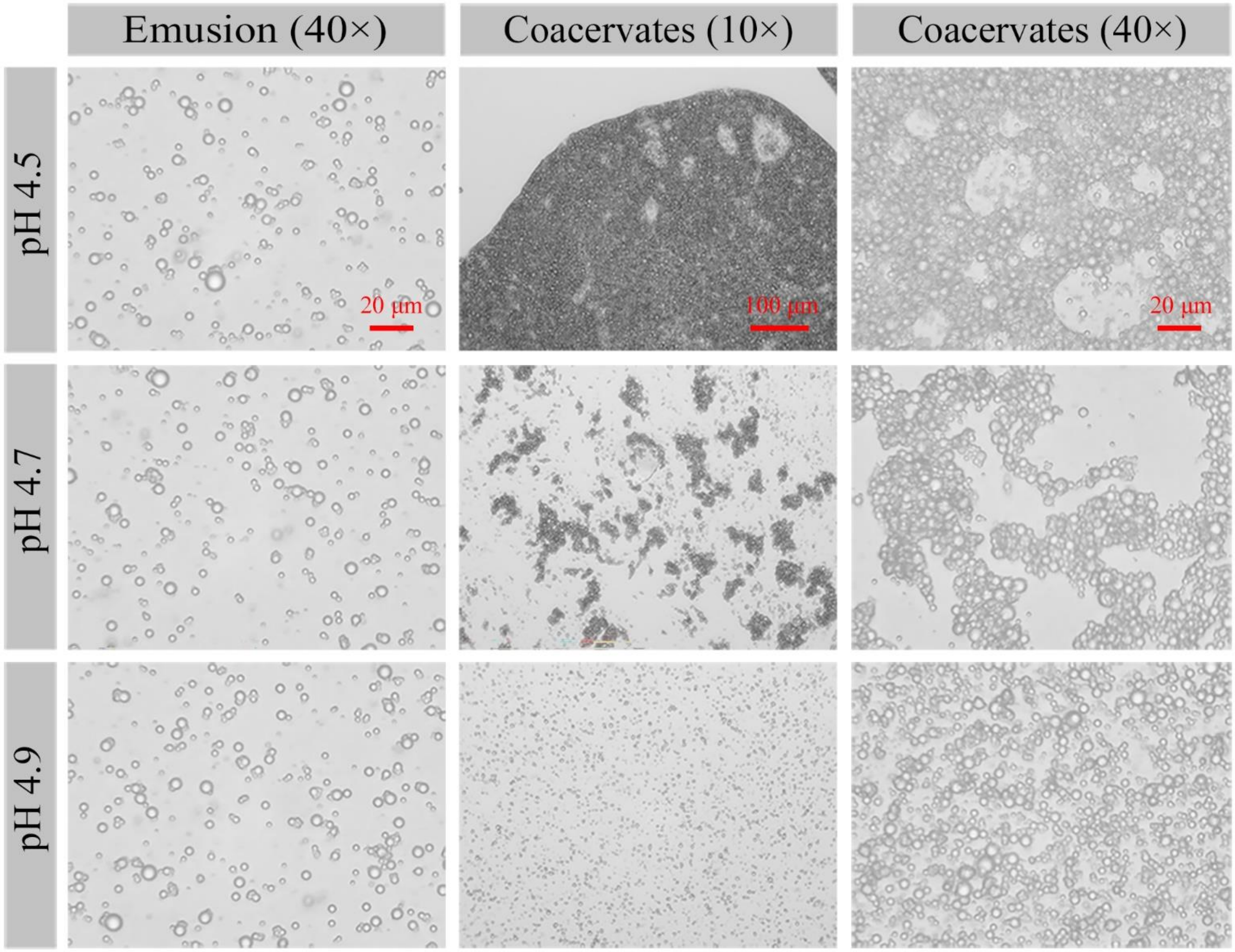

Supplementary Figure 2. Effect of pH on the formation of fish oil-loaded CFG-stabilized emulsions and fish oil@CFG-SHMP complex coacervates. The samples were photographed by an upright optical microscope with 10× and 40× objectives. The gelatin concentration was 80 mg/mL.

Supplementary Figure 3.

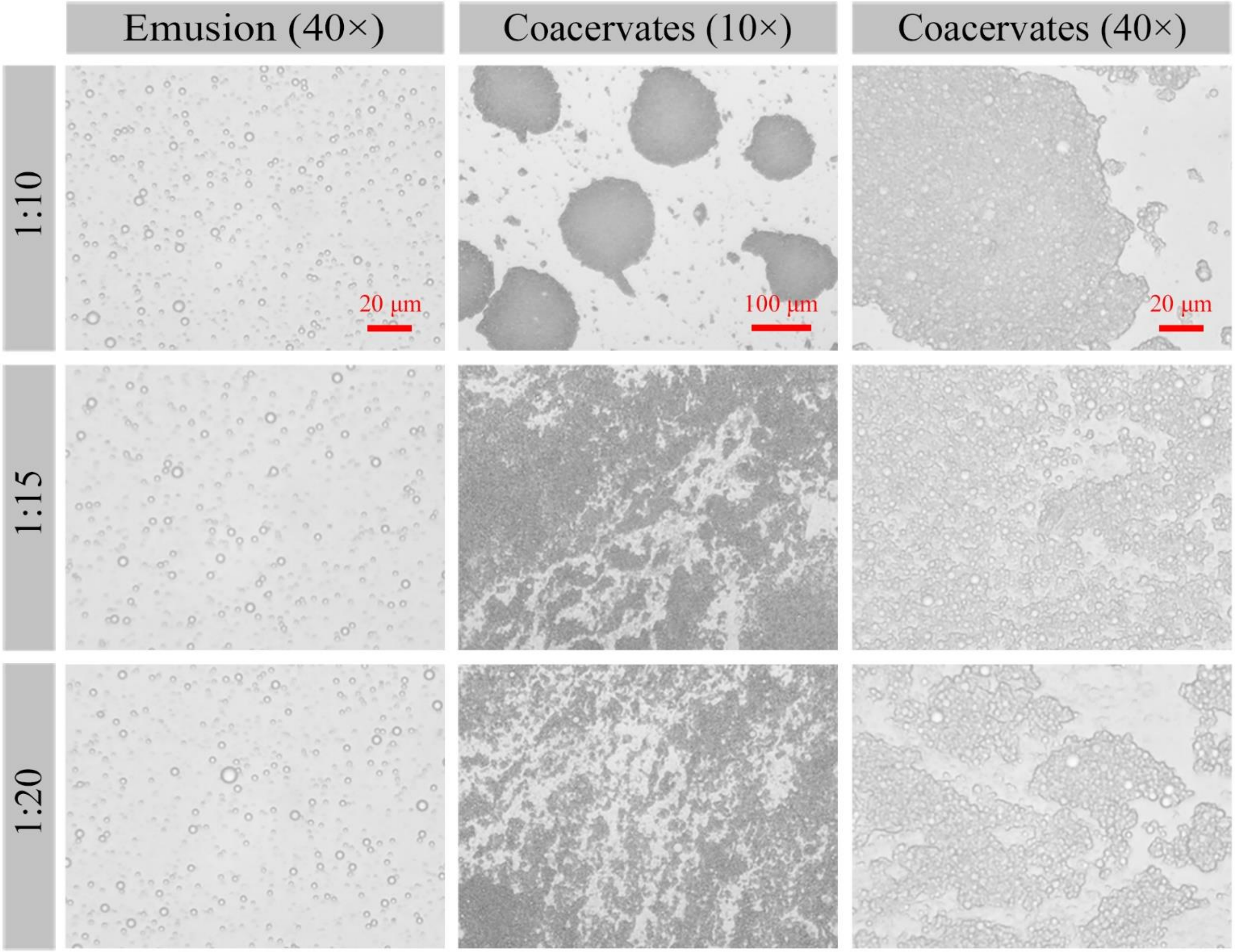

Supplementary Figure 3. Effect of SHMP:gelatin mass ratio on the formation of fish oil-loaded CFG-stabilized emulsions and fish oil@CFG-SHMP complex coacervates. The samples were photographed by an upright optical microscopy with 10× and 40× objectives. The gelatin concentration was 80 mg/mL.

Supplementary Figure 4.

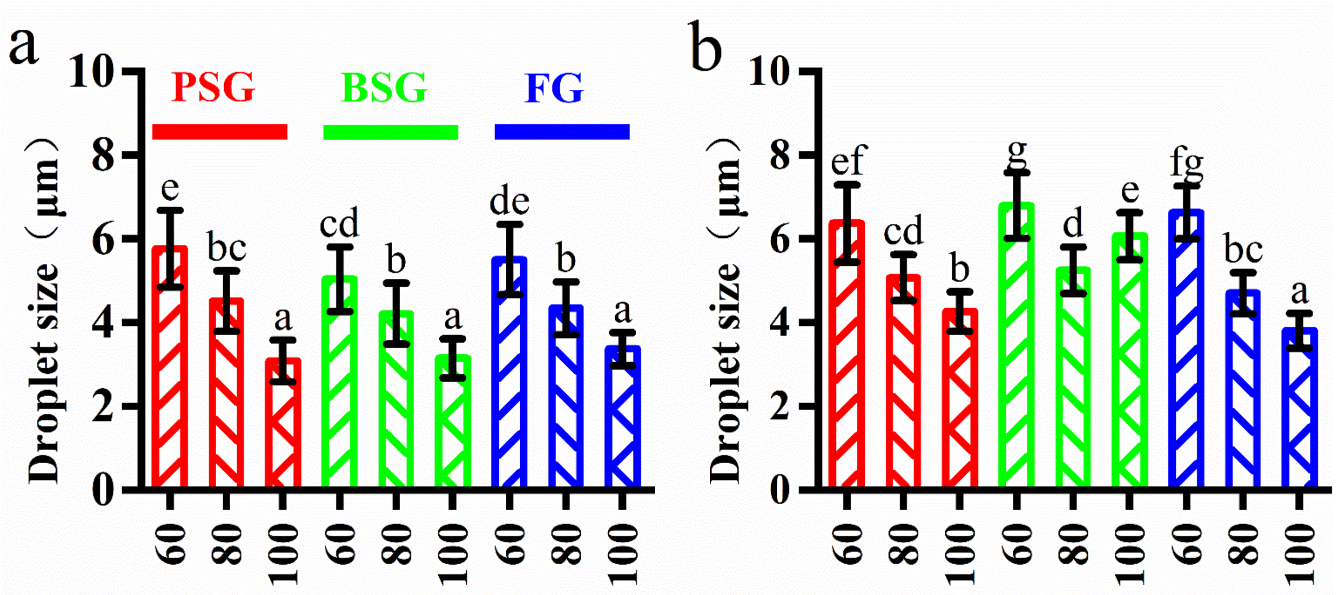

Supplementary Figure 4. The average sizes of the fish oil droplets in the emulsions (A) and powders (B). Error bars refer to standard deviation of the mean.

Supplementary Figure 5.

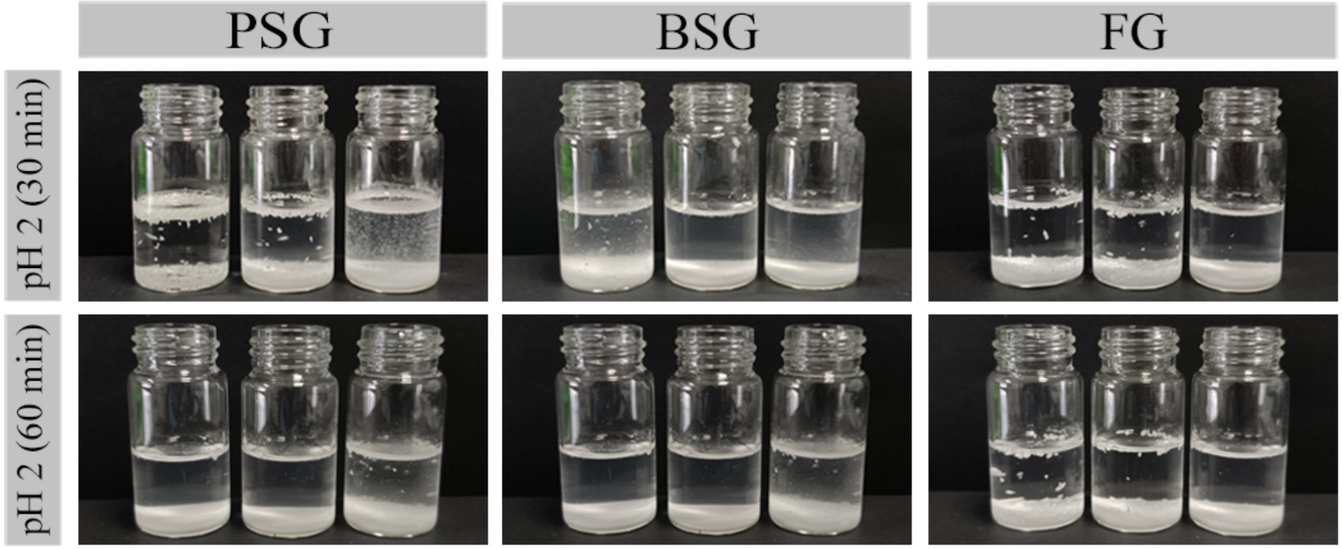

Supplementary Figure 5. Digital camera images of the fish oil@gelatin-SHMP@SSOS powders in phosphate buffered saline (PBS) solution (pH 2.0) at different incubation times (30 min and 60 min). The gelatin concentrations from left to right in each image were 60, 80, and 100 mg/mL.

Supplementary Figure 6.

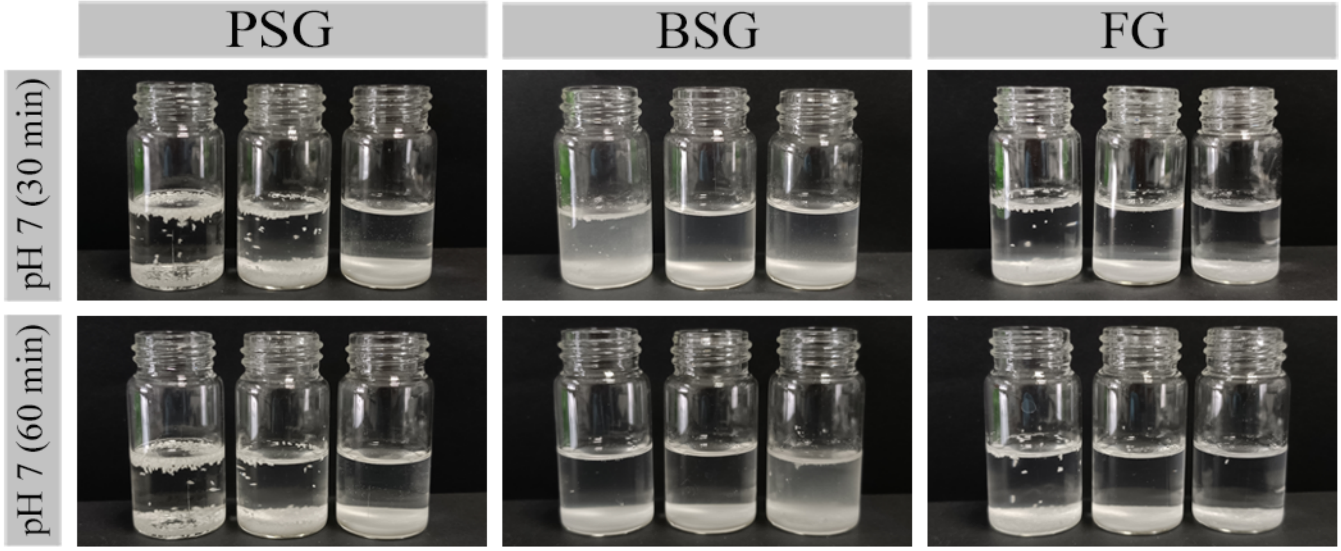

Supplementary Figure 6. Digital camera images of the fish oil@gelatin-SHMP@SSOS powders in PBS solution (pH 7.0) at different incubation times (30 min and 60 min). The gelatin concentrations from left to right in each image were 60, 80, and 100 mg/mL.

Supplementary Figure 7.

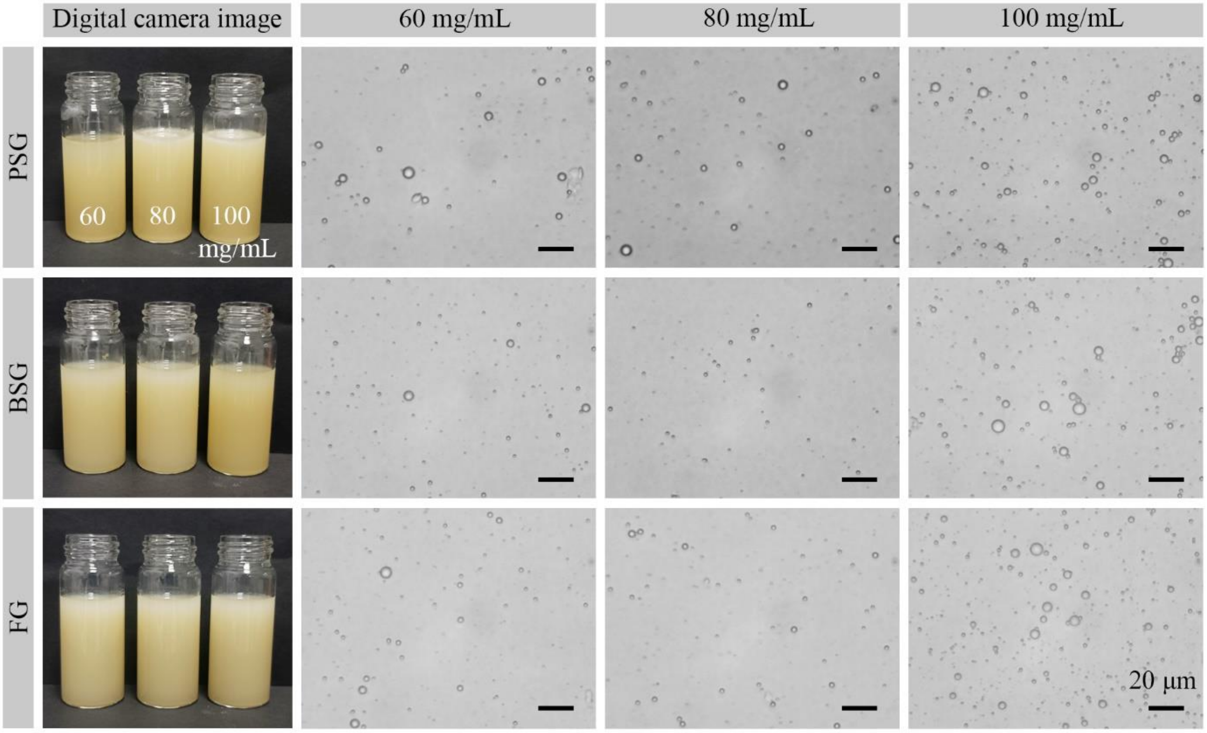

Supplementary Figure 7. Digital camera and optical microscopy observation of the fish oil@gelatin-SHMP@SSOS powders at different gelatin concentrations (60, 80, and 100 mg/mL) after the gastrointestinal and small intestinal phases. Scale bar indicates 20 µm.
